# Proposing Bromo-Epi-Androsterone for Host-Directed Therapy Against Tuberculosis

**DOI:** 10.3390/pathogens14111179

**Published:** 2025-11-18

**Authors:** Coad Thomas Dow, Liam Obaid

**Affiliations:** 1 McPherson Eye Research Institute, University of Wisconsin-Madison, Madison, WI 53705, USA; 2 Keck School of Medicine, University of Southern California, Los Angeles, CA 90089, USA; lobaid@usc.edu

**Keywords:** *Mycobacterium tuberculosis*, Mtb, DHEA, bromoepiandrosterone, BEA, immunosenescence, MDR-TB, murine model, host-directed immuno-therapy

## Abstract

Bromoepiandrosterone (BEA), a synthetic analog of the adrenal steroid DHEA, holds promise as a host-directed therapy for both active and latent tuberculosis (TB). Unlike DHEA, BEA lacks hormonal side effects yet retains potent immunomodulatory activity. It promotes a Th1-skewed immune response by enhancing interferon-γ (IFN-γ) and tumor necrosis factor-α (TNF-α), critical cytokines for macrophage activation and intracellular control of *Mycobacterium tuberculosis* (Mtb), while suppressing Th2 cytokines such as IL-4. BEA also inhibits 11β-hydroxysteroid dehydrogenase-1, lowering intracellular cortisol levels and reversing the local immunosuppression commonly seen in TB. These features enable BEA to restore immune competency in TB-infected tissues. In murine TB models, BEA halted bacterial growth, reduced pulmonary inflammation, and synergized with standard anti-TB drugs to enhance bacterial clearance. Additionally, DHEA and its analogues have demonstrated direct antimycobacterial activity, likely by interfering with Mtb mycolic acid synthesis, a property BEA is believed to share. For latent TB, BEA’s ability to sustain Th1-mediated immunity and counteract immune suppression could help maintain latency and prevent reactivation, especially in immunocompromised individuals. By boosting immune surveillance and potentially contributing to bacillary clearance, BEA offers a unique adjunctive approach that complements existing TB treatments without contributing to drug resistance. Its dual function, an immune modulator and antimicrobial agent, supports its use across the TB disease spectrum. These properties position BEA as a novel candidate for host-directed therapy aimed at improving outcomes in both drug-sensitive and drug-resistant TB, as well as therapies aimed at enhancing long-term containment of latent infection.

## 1. Introduction

Tuberculosis (TB) remains a leading cause of infectious disease mortality despite being both preventable and curable. According to the 2024 WHO Global Tuberculosis Report, 8.2 million people were newly diagnosed with TB in 2023, and an estimated 10.8 million people contracted TB that year: TB again surpassed COVID-19 as the world’s deadliest infectious disease, causing about 1.25 million deaths [1]. The burden is concentrated in thirty mostly low- and middle-income countries—India, Indonesia, China, the Philippines, and Pakistan account for over half of incident cases [2]. Major drivers include under-nutrition, HIV infection, diabetes, alcohol use, and smoking, and the report notes that funding for prevention, diagnostics, and treatment remains well below the global target [2]. Multidrug-resistant and rifampicin-resistant (MDR/RR) TB continues to threaten progress; approximately 400,000 new MDR/RR-TB cases and 150,000 deaths occurred in 2023, and treatment success remains below 70% in many settings [3]. These gaps underscore the urgent need for host-directed therapies, shorter treatment regimens, and effective vaccines to improve immune control of *Mycobacterium tuberculosis* [2,3]. Moreover, HIV remains a potent risk factor for TB reactivation, compounding disease burden in regions with high HIV prevalence [4].

## 2. Ageing, Immunosenescence, and TB Susceptibility

The global population is ageing; people aged ≥65 years now represent a rapidly growing demographic, and TB incidence and mortality are disproportionately high in this group [5]. Elderly patients often present with frailty, malnutrition, comorbidities, and polypharmacy [5]. These factors are compounded by immunosenescence, the age-related decline in immune function. Immunosenescence involves both quantitative and qualitative impairments in innate and adaptive immunity: there is thymic involution with reduced output of naive T cells, accumulation of senescent memory T cells, and a shift in CD4^+^ T-helper responses toward Th2 at the expense of Th1 [6]. Senescent T cells lose proliferative capacity and secrete pro-inflammatory cytokines (senescence-associated secretory phenotype, SASP), producing a state of chronic low-grade inflammation (inflammaging) [6]. In innate compartments, neutrophils and macrophages exhibit reduced chemotaxis, phagocytosis, and antigen presentation [6]. Together, these alterations signify a diminished immune capacity; the loss of naive lymphocytes, expansion of dysfunctional memory cells, and chronic pro-inflammatory milieu leave older adults with a blunted ability to respond to novel pathogens and to maintain effective cellular immunity against Mtb [6].

Clinical observations corroborate these immunobiological changes. Older adults display declining lung function and respiratory muscle strength, which impair mucociliary clearance and promote aspiration [5]. They also have diminished repair capacity and are more likely to carry comorbidities (e.g., diabetes) that further compromise immunity [5]. Consequently, most active TB disease in older adults is due to reactivation of latent infection rather than recent transmission [5]. In addition, the efficacy of BCG and other vaccines is reduced in the elderly because antibody production, class-switch recombination, and generation of long-lived plasma cells all decline with age [6]. Immunosenescence thus constitutes a major risk factor for TB, highlighting the need for age-tailored vaccines and immune-boosting therapies.

### 2.1. Immune Commitment to Persistent Viral and Environmental Factors

Age-related immune decline is not driven by senescence alone but also by the cumulative burden of persistent antigenic and environmental stressors. These pressures continuously engage the immune system, accelerating exhaustion and diminishing its ability to respond to new infections such as Mtb [7]. The most prominent of these influences are chronic viral infections, particularly human cytomegalovirus (HCMV) and human immunodeficiency virus (HIV), followed by metabolic, nutritional, and environmental factors that perpetuate low-grade inflammation.

#### 2.1.1. Viral Infections

Among chronic infections, HCMV plays an underappreciated but central role in immunosenescence. As a ubiquitous β-herpesvirus, HCMV establishes lifelong latency and is highly prevalent in low-resource settings where seropositivity often exceeds 90% by adolescence [8]. Persistent HCMV replication drives “memory inflation”—massive expansion of virus-specific CD8^+^ T cells that overtake the T-cell repertoire, contract the naïve pool, and increase expression of senescence and exhaustion markers such as CD57 and PD-1 [7,8]. This state of chronic immune activation elevates circulating type I interferons and IL-10 homologs that suppress macrophage activation and disrupt granuloma integrity [8,9]. The consequence is impaired containment of intracellular pathogens, including *M. tuberculosis*.

Epidemiological data substantiate this mechanistic link. In a South African infant cohort, those with HCMV-specific IFN-γ responses had more than double the odds of developing TB disease compared with HCMV-negative peers (adjusted OR 2.2, 95% CI 1.02–4.83) [8]. A 2023 systematic review and meta-analysis encompassing >38,000 participants confirmed that HCMV-infected individuals had threefold higher odds of active TB (pooled OR 3.20, 95% CI 2.18–4.70), rising to fourfold in those with the highest antibody titers [9]. These findings establish HCMV as a significant, if underrecognized, co-factor in global TB susceptibility.

HIV infection likewise exemplifies chronic immune commitment leading to premature immune ageing. Persistent viral replication depletes CD4^+^ T cells, damages lymphoid architecture, and induces systemic inflammation and expansion of senescent T-cell subsets [7,10]. The continuous requirement to suppress HIV replication accelerates immune exhaustion, diminishing responsiveness to *Mtb* antigens and compounding the risk of TB reactivation [4,10,11]. In this way, both HCMV and HIV illustrate how lifelong viral surveillance diverts and erodes immune resources essential for TB control.

#### 2.1.2. Environmental and Metabolic Influences

Beyond viral infections, several external factors further amplify immunosenescence and TB risk. Chronic under-nutrition, air-pollution exposure, and metabolic disorders such as diabetes sustain oxidative stress and systemic inflammation [5,6]. Endocrine perturbations—including sustained psychosocial stress or prolonged glucocorticoid exposure—elevate the cortisol–DHEA ratio, promoting immunosuppression and inflammaging [12,13,14,15]. These environmental and metabolic stressors thus act synergistically with chronic infections to shape the aged, inflammation-prone immune phenotype.

## 3. Immunosenescence and the Role of DHEA Decline

Beyond these chronic viral antigenic exposures, immunosenescence also arises from endocrine alterations; among these, the age-related decline of the adrenal steroids dehydroepiandrosterone (DHEA) and its sulfated form DHEA-S (hereafter collectively referred to as DHEA) plays a central role. DHEA is one of the most abundant C-19 adrenal steroids in circulation, peaking in early adulthood and decreasing steadily thereafter. Classic studies more than six decades ago showed that mean plasma DHEA levels in normal adult males declined from ~48 μg/100 mL at age 20–29 to ~2 μg/100 mL by age 80–89, demonstrating a nearly twenty-fold decrease with age [16]. This natural reduction shifts the cortisol–DHEA ratio because cortisol levels remain relatively stable across life; the loss of DHEA’s immunomodulatory counterbalance to glucocorticoid activity leads to chronic low-grade inflammation and diminished immune resilience.

DHEA exerts broad immunomodulatory actions on both innate and adaptive immunity. It enhances natural killer (NK)-cell cytotoxicity and macrophage phagocytic capacity, functions that tend to wane as DHEA levels fall [12,13]. At the adaptive level, DHEA promotes Th1-type responses by up-regulating interleukin-2 (IL-2) and interferon-γ (IFN-γ) and suppressing Th2 cytokines [13]; its decline therefore predisposes older individuals toward a Th2-skewed profile, compromising defense against intracellular pathogens including *Mycobacterium tuberculosis*.

Endocrine studies indicate that a higher cortisol–DHEA ratio, reflecting low DHEA availability, is associated with age-related declines in immune function and increased susceptibility to infection [12,14]. Elevated pro-inflammatory cytokines such as IL-6 and soluble IL-6 receptor levels are commonly found in chronic inflammatory diseases and frailty [15].

Clinically, age-related reduction in DHEA correlates with poorer vaccine responses, higher risk of respiratory infections, and frailty in older adults [15,17]. Observational studies show that lower DHEA levels are associated with frailty status in both men and women [15]. Some intervention trials have examined DHEA supplementation in elderly or immunocompromised populations (including HIV-positive patients) [18,19]. Small placebo-controlled studies report partial restoration of Th1 cytokines; modest improvements in mood and well-being; and, in some cases, enhanced NK-cell activity. However, the results vary by dose, sex, and baseline immune status [14,18,19]. Importantly, translational research is hampered by species differences: rodents do not synthesize adrenal DHEA because their adrenals lack the enzyme P450c [20], limiting extrapolation of murine findings to humans. Moreover, a randomized controlled trial in HIV-positive adults found no antiviral benefit and only modest improvements in quality of life [14]. These mixed results underscore the need for further rigorous trials to determine whether host-directed therapy with DHEA can safely enhance immune resilience in ageing populations or chronic infections.

In all, the decline of DHEA with age is a key hormonal driver of immunosenescence. The loss of this steroid’s immunomodulatory effects shifts the cortisol–DHEA balance, fosters chronic low-grade inflammation, and weakens both innate and adaptive immune responses. Strategies that restore or mimic DHEA activity, through endocrine therapy, represent promising host-directed approaches to bolster immune defenses in older adults and those with chronic infections.

## 4. History of Bromoepiandrosterone (BEA, Originally Known as HE2000) as an Anti-Infective Agent

Early work identified dehydroepiandrosterone (DHEA) and its halogenated analogue bromoepiandrosterone (BEA) as inhibitors of Epstein–Barr virus-induced transformation of human lymphocytes; BEA was reported to be about 60-fold more potent than DHEA as a glucose-6-phosphate dehydrogenase inhibitor and more effective in blocking virus-driven DNA synthesis [21]. Subsequent feline studies highlighted a broader antiviral spectrum, demonstrating that BEA suppressed feline immunodeficiency virus replication and modulated viraemia [22].

BEA also exhibits pronounced antiparasitic effects. Low-micromolar concentrations of BEA trigger externalization of phosphatidylserine on ring-stage *Plasmodium falciparum*–infected erythrocytes, enabling non-opsonic phagocytosis by monocytes [23]. Beyond malaria, brominated BEA potently inhibits *Trypanosoma cruzi* glucose-6-phosphate dehydrogenase through a halogen-bond interaction [24].

In TB models, BEA restores Th1 immunity and enhances bacterial clearance [25]. A water-miscible formulation of BEA lowers 11β-hydroxysteroid dehydrogenase-1 (5β-HSD1) and corticosterone, reactivating protective immunity [26]. In diabetic TB models, BEA decreases 11β-HSD1 and glucocorticoids; increases 11β-HSD2; and reduces hyperglycemia, steatosis, and bacillary burden [26]. BEA also limits non-productive lung inflammation and enhances interferon-γ–dependent immunity in a cystic-fibrosis model [27].

**Figure 1:**
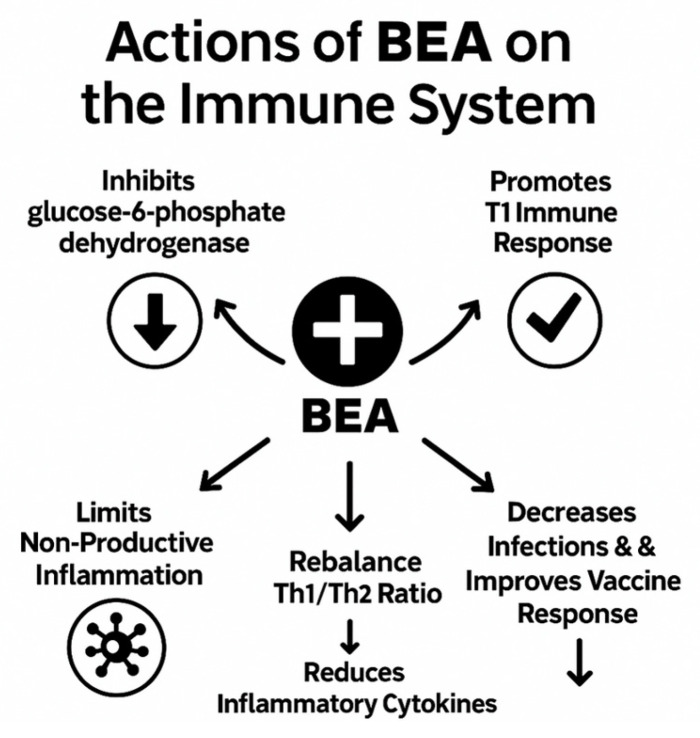
BEA effects on immune function.

Mechanistic insights suggest that DHEA/BEA may bind the *Mycobacterium tuberculosis* enoyl-acyl carrier protein reductase (InhA) because of structural homology with human 11β-HSD1; this interaction increases temporary pro-inflammatory cytokine production and may provide a direct antimycobacterial effect [25]. Clinical data remain limited but promising: the pilot study in acute falciparum malaria found that adjunctive BEA therapy accelerated parasite clearance and fever resolution without serious adverse events [23]. In AIDS patients, a double-blind trial showed that BEA significantly reduced the incidence of TB and other opportunistic infections compared with placebo, with good tolerability [27]. In HIV-1–infected individuals, BEA increased dendritic and CD8 T-cell numbers, downregulated inflammatory gene transcripts, and reduced viral load [28,29]. More recently, BEA’s potent anti-inflammatory effects have been proposed as a potential therapy for neuroinflammatory conditions such as post-traumatic stress disorder [30] (Figure 1). Collectively, these findings illustrate BEA’s unique immuno-metabolic profile and support further evaluation as an adjunct therapy for TB and other infections.

## 5. Rationale for Bromoepiandrosterone (BEA) for Tuberculosis

**Figure 2:**
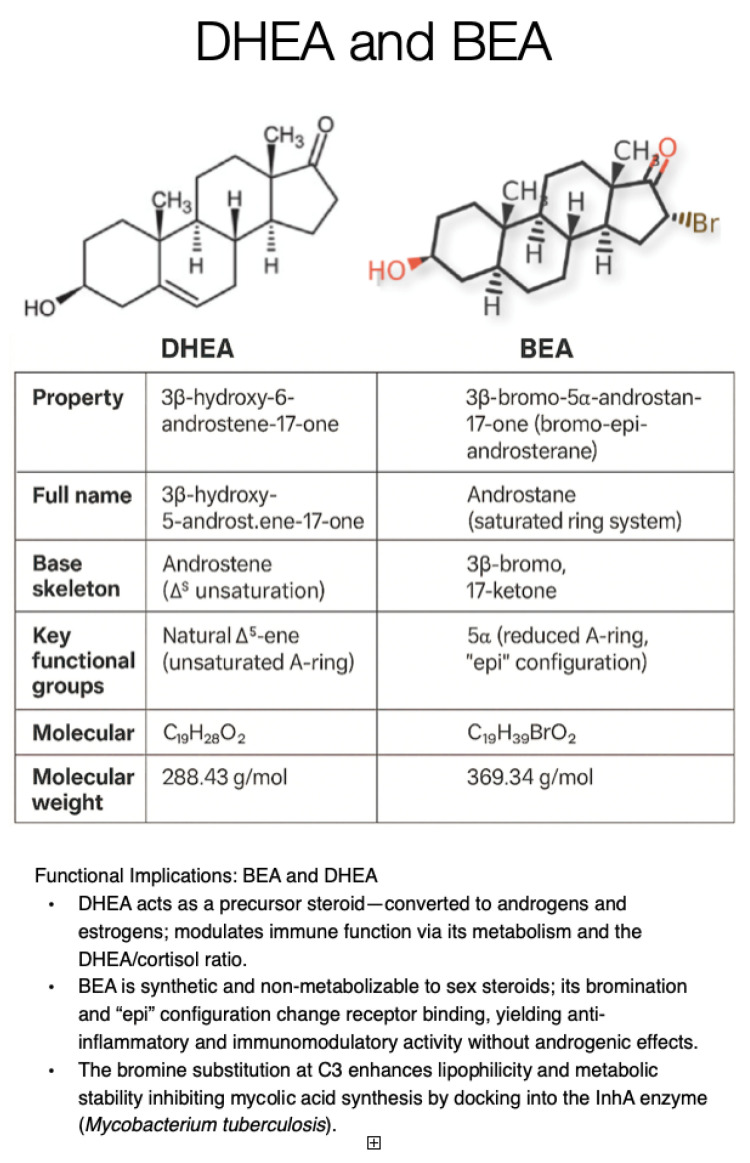
Comparison of DHEA and BEA.

Bromoepiandrosterone (BEA) is a synthetic derivative of the adrenal steroid DHEA designed to harness DHEA’s immune benefits without its hormonal side effects. Unlike DHEA, BEA does not convert to sex steroids, making it safer for therapeutic use [28]. BEA exerts potent immunomodulatory effects: it skews immune responses toward a protective Th1 profile, increasing key cytokines such as interferon γ (IFN-γ) and tumor necrosis factor α (TNF-α) that activate macrophages, while lowering Th2/immunosuppressive signals (e.g., IL-4) [29]. DHEA itself has anti-glucocorticoid properties and promotes Th1 cytokine production in human cells, properties that BEA was designed to amplify [25]. BEA also counteracts the excess glucocorticoids induced during TB infection by inhibiting 11β-hydroxysteroid dehydrogenase 1, thereby reducing local cortisol levels and reactivating anti-TB immunity [26]. In addition to modulating host immunity, BEA (like DHEA) may directly impede *Mycobacterium tuberculosis*: DHEA displays a dose-dependent microbicidal effect on Mtb in vitro, likely by docking into the InhA enzyme to inhibit mycolic-acid synthesis, a mechanism BEA is presumed to share [26] (Figure 2).

These properties translate to improved infection control in vivo. In murine TB models, BEA treatment halted bacterial growth and boosted the expression of IFN-γ, TNF-α, and inducible nitric oxide synthase while reducing IL-4 levels [25]. Such immune enhancement reduces mycobacterial burden: treated mice display significantly lower lung bacterial loads and less pneumonia compared with controls [25]. When used as an adjunct to standard TB chemotherapy, BEA accelerates bacterial clearance [25], suggesting it could shorten treatment duration or improve outcomes in drug-resistant TB. Because BEA acts on host pathways, its effectiveness is not diminished by Mtb drug resistance.

BEA may also benefit individuals with latent TB infection or comorbidities. Latent TB is kept in check by sustained cell-mediated immunity; BEA’s ability to maintain a strong Th1 response and activate macrophages could help preserve this containment. By suppressing local immunosuppressive corticosteroid production in the lung and enhancing immune surveillance, BEA may reduce the risk of latent TB reactivation and aid in the eradication of residual bacilli [27]. These effects could be particularly valuable in persons with weakened immunity (such as those with diabetes or HIV co-infection), where BEA could compensate for immunodeficiencies [25,26]. Overall, the dual immunomodulatory and antimicrobial actions of BEA provide a strong biological rationale for its use in both active and latent TB, offering a potential adjunctive therapy that could improve treatment efficacy and patient outcomes.

## 6. BEA Activity in Murine Models of Tuberculosis

Although mice do not produce appreciable levels of dehydroepiandrosterone (DHEA) due to absent or minimal adrenal 17,20-lyase activity [20], they remain a valuable model system for investigating the host-directed effects of synthetic analogs such as 16α-bromoepiandrosterone (BEA). The mechanistic basis for this lies in the fact that BEA does not require metabolic conversion into sex steroids to exert biological activity. Instead, BEA functions as an immunomodulatory agent acting through conserved metabolic and inflammatory pathways that are shared across mammalian species, including rodents.

Murine studies conducted by Hernández-Pando and colleagues demonstrated that BEA restores Th1-mediated immune activity, characterized by increased interferon-γ (IFN-γ), tumor necrosis factor-α (TNF-α), and inducible nitric oxide synthase (iNOS) expression. These effects were associated with enhanced bacterial clearance when BEA was used alone or in conjunction with standard TB chemotherapy [25]. Additional work showed that BEA limits non-productive inflammation while preserving protective immune responses in the lung, in part by reducing nitric oxide overproduction by macrophages [27]. More recently, in a murine model of TB complicated by type 2 diabetes, BEA was shown to modulate local glucocorticoid metabolism through downregulation of 11β-hydroxysteroid dehydrogenase type 1 (11β-HSD1) and upregulation of 11β-HSD2, thereby reducing hyperglycemia, steatosis, and TB-related pulmonary pathology [26]. Together, these findings highlight BEA’s ability to rebalance host immunity by dampening harmful inflammation while augmenting pathogen-clearing responses.

These outcomes underscore why the murine model provides meaningful insights into BEA’s host-directed mechanism despite the species’ lack of endogenous DHEA production. Because BEA is brominated at the 17-position, it cannot serve as a precursor to sex hormones; instead, it acts directly on immune and metabolic checkpoints. Thus, the beneficial effects observed in mice derive from species-independent immunoregulatory pathways, reinforcing the translational relevance of these findings to human TB.

## 7. Challenging the Murine Model of MDR-TB

**Figure 3:**
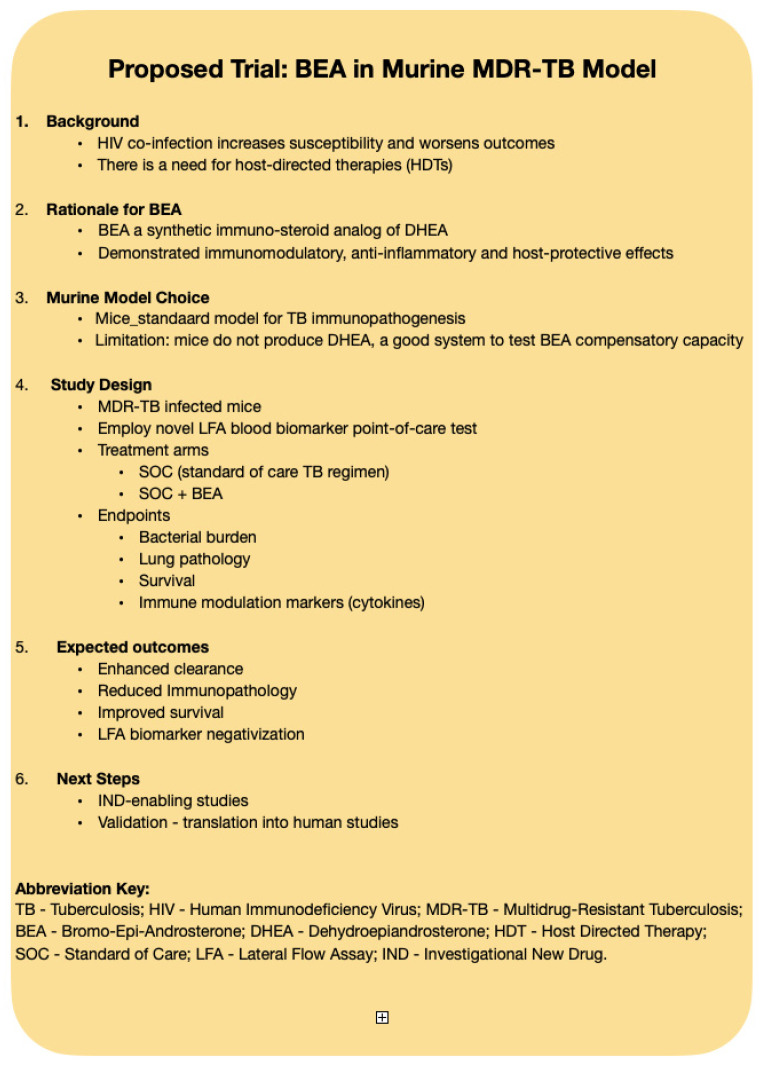
Proposed Study with BEA for Animal Model of MDR-TB.

MDR-TB represents the sharpest edge of the TB epidemic. Despite progress with newer TB drugs, there remains an urgent need for adjunctive strategies that can modulate the host response, improve survival, and limit tissue destruction. This is the rationale for proposing a trial of bromo-epi-androsterone (BEA) in the murine model of MDR-TB (Figure 3). The murine system is intentionally stringent: unlike humans, mice do not produce endogenous DHEA, creating a setting in which any protective effect of BEA must emerge against a baseline of deficiency. Infection with MDR strains ensures that antibiotic efficacy is intrinsically limited, making this model a tough but fair test of whether BEA can meaningfully alter disease course. This study design contrasts standard-of-care antibiotic therapy with and without BEA, using endpoints that include bacterial burden, lung pathology, survival, and immune markers. In this way, the experiment directly addresses whether BEA can provide an additive benefit on top of optimized drug therapy.

## 8. Summary

If BEA use results in even modest improvements, e.g., fewer colony-forming units in the lungs, reduced granulomatous damage, or better survival curves, the implications would be substantial. Demonstrating host-directed benefit in a model where antibiotics alone fail to sterilize TB infection would establish BEA as a candidate adjunctive therapy for MDR-TB, as well as potentially for HIV/TB co-infected patients, who are at the highest risk of death. Success in this stringent preclinical setting would justify the transition to IND-enabling studies, guide dose selection, and support clinical testing. In short, a favorable outcome in the murine MDR-TB model would not only validate BEA’s mechanism of action but also provide hope for tackling the most intractable form of TB, where new therapeutic strategies are most urgently needed.

## Data Availability

Not applicable.
